# Development of the body image self-rating questionnaire for breast cancer (BISQ-BC) for Chinese mainland patients

**DOI:** 10.1186/s12885-017-3865-5

**Published:** 2018-01-04

**Authors:** Kaina Zhou, Xiaole He, Lanting Huo, Jinghua An, Minjie Li, Wen Wang, Xiaomei Li

**Affiliations:** Xi’an Jiaotong University Health Science Centre, 76 Yanta West Road, Xi’an, Shaanxi 710061 China

**Keywords:** Body image, Breast cancer, China, Mixed methods

## Abstract

**Background:**

Body image is a complex post-treatment concern among female patients with breast cancer, and various tools have been developed and applied to measure this multifaceted issue. However, these available tools were developed in other countries and only a few have been modified into Chinese versions. Furthermore, body-image evaluation instruments that are specific to Chinese mainland female patients with breast cancer have not been devised yet. Therefore, we developed the Body Image Self-rating Questionnaire for Breast Cancer for Chinese mainland female patients with breast cancer.

**Methods:**

We performed two rounds of the Delphi technique and a cross-sectional pilot survey. Items were selected using a Likert scale (1–5) to determine ratings of importance (i.e., the significance of the item from experts’ perspective; coefficients of variation ≤0.25), internal consistency reliability (Cronbach’s α ≥ 0.70), convergent validity (hypothesized item-subscale correlations ≥0.40), and discriminant validity (stronger correlations of the item with the hypothesized subscale than for other subscales). All decisions on items were made based on statistical analysis results, experts’ recommendations, and in-depth discussion among researchers.

**Results:**

Twenty-five eligible experts completed the two Delphi rounds (mean age: 42.20 ± 8.90 years). Over half the experts were professors (56%, *n* = 14) or worked as clinical staff (68%, *n* = 17). Twenty (mean age = 49.55 ± 10.01 years) and 50 patients (mean age = 48.44 ± 9.98 years) completed the first- and second-round survey, respectively. Over half the patients had a tertiary education level, were married, and were employed. Regarding the revised questionnaire (comprising 33 items across seven subscales), the expert panelists’ ratings of each item met the criteria (Kendall’s W = 0.238, *p* < .001). Five subscales had a Cronbach’s α value over 0.60 (range: 0.62–0.69) and two subscales were over 0.80 (range: 0.84–0.88). All items satisfied the criteria for convergent and discriminant validity.

**Conclusions:**

The findings of this study provide evidence of a suitable tool for body image evaluation among Chinese mainland female patients with breast cancer. Studies with larger sample sizes should be conducted to validate this questionnaire in this patient population.

**Electronic supplementary material:**

The online version of this article (10.1186/s12885-017-3865-5) contains supplementary material, which is available to authorized users.

## Background

Body image reflects a multifaceted concept involving perceptions, thoughts, emotions, and behaviors regarding one’s appearance and physical functioning [1]. It can be influenced by physical, psychological, and social functioning changes resulting from breast cancer treatment [2]; from surgeries leaving disfigurations, scars, sensation alteration, and shoulder/arm functioning impairments [3–5]; chemotherapy resulting in hair loss and weight gain [6, 7]; radiotherapy leading to skin discoloration, dermatitis, and soreness of the treated area [8]; and hormonal therapies causing premature menopause, body pains, and vasomotor symptoms [9]. Disturbed body image is considered the key contributor of overall negative psychological states as well as poorer health-related quality of life [4, 10–12]. Findings of a systematic review suggest that body image has become a complex posttreatment concern for female patients with breast cancer [13].

In the context of breast cancer, the construct of body image is multidimensional. There are three characteristics of body image concept in patients with breast cancer: affective (feeling attractive and feminine), behavioral (avoiding people due to appearance), and cognitive (satisfaction with scars or appearance) [14]. Additionally, body image after breast cancer also includes the characteristics of the mental image of one’s body, attitude about appearance and health state, and sexual functioning [15]. A theoretical framework regarding body image in female patients with breast cancer, who underwent breast reconstruction, specifically involves aspects of perception, cognition, behavior, and emotion, which all link to the function of the body following breast cancer diagnosis and treatment [16].

Although the complexity of body image in patients with breast cancer has been documented, various tools have been developed and applied to measure this complex issue among this patient population: the Body Image Avoidance Questionnaire (BIAQ) [17], the Body Image Scale (BIS) [14] and its modified Chinese version [18], the Body Image after Breast Cancer Questionnaire (BIBCQ) [19], the Body Image and Relationships Scale (BIRS) [20], the Sexual Adjustment and Body Image Scale (SABIS) [21], the Breast and Body Image Scale (BBIS) [22], and the Body Image Psychological Inflexibility Scale (BIPIS) [23]. These instruments evaluate body image from specific facets of body image characteristics after breast cancer, and some of them have been adapted into and validated in other language versions [24–29].

However, few of these tools have considered the comprehensive characteristics of body image after breast cancer (e.g., affective, behavioral, cognitive, attitude, sexual functioning, perception, and emotion) in one questionnaire [17, 20, 21, 23]. Since body image is people’s perception of the aesthetics or sexual attractiveness of their own body [30], previously developed tools seem to place less emphasis on measuring body image from the patients’ viewpoint. Although these available tools were developed in other countries, and a few were modified into Chinese versions [18, 24], a body-image evaluation instrument that is specific to Chinese mainland female patients with breast cancer has not been developed yet.

When developing new instruments, the Delphi technique is the most widely used method. It aims to obtain reliable consensus on a given topic through two to four consecutive rounds of a questionnaire survey with 10 to 30 experts [31]. It is extremely useful in conditions where individual judgments must be tapped and combined to address a lack of agreement or incomplete knowledge. However, the Delphi technique alone may be somewhat inadequate in developing patient self-rated questionnaires, in that it lacks the feedback of the target population [32]. Accordingly, conducting a pilot cross-sectional survey at the same time as the Delphi rounds might provide useful supplementary information from the target population, which can be further used in instrument development.

Drawing on the characteristics and theoretical framework regarding body image in the breast cancer context, we developed the Body Image Self-rating Questionnaire for Breast Cancer (BISQ-BC) in Chinese mainland patients with breast cancer via using the simultaneous application of the Delphi technique and a pilot cross-sectional survey. The study findings will provide evidence for body image evaluation in practice among patients with breast cancer.

## Methods

### Item pool establishment

The item pool was devised based on the characteristics of body image concepts [14, 15] and the described theoretical framework [16]. The devised items concerned body-image-related self-cognition as well as the change in behavior, shoulder/arm functioning, sexual activity, role, and psychological and social status. Additional items were drafted using existing questionnaires, including the BIAQ [17], the BIS [14], the BIBCQ [19], the BIRS [20], the SABIS [21], the BBIS [22], and the BIPIS [23]; from a review of literature on body image of patients with breast cancer [13]; and from other reports on body image of female patients with breast cancer [4, 33, 34]. All drafted items were revised through in-depth discussion among the research team members to fit with the Chinese culture and breast cancer care settings. In terms of person-centered holistic nursing care principles [35], the items were designed using a self-rating format and adhered to the viewpoint of patients with breast cancer. Since the target population was Chinese mainland female patients with breast cancer, all items were presented in Mandarin.

### Delphi technique

The experts of the Delphi panel were either recruited within our own network and approached by a researcher or they received an invitation to participate from one of the recruited experts (i.e., snowball sampling). They were clinicians or nurses working with patients with breast cancer and scholars majoring in breast cancer nursing research. Twenty-five experts were recruited from comprehensive universities and tertiary hospitals in Xi’an (*n* = 12), Peking (*n* = 3), Shanghai (*n* = 3), Guangzhou (*n* = 3), Sichuan (*n* = 2), and Hunan (*n* = 2). Several reminders were sent out during each round requesting that non-responding experts complete the questionnaire within two weeks. Additionally, after completing the Delphi procedure, all experts received a book voucher as compensation.

**Fig. 1:**
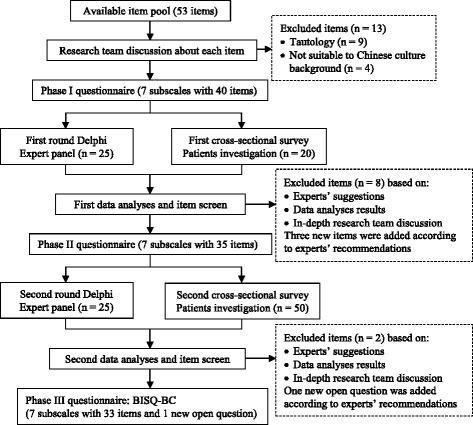
Flow chart of items selection of the Body Image Self-rating Questionnaire for Breast Cancer (BISQ-BC)

The Delphi questionnaire comprised two sections. The first section asked for experts’ personal information (i.e., age, professional title, occupation, and education level). The second section included the complete BISQ-BC (phase I questionnaire; see Fig. 1) with detailed descriptions of each subscale and item. Experts were asked to rate each item on a closed five-point Likert scale (1 = *not important*, 5 = *very important*) with additional blanks to allow them to fill in revision comments. At the end of the questionnaire, experts could also provide opinions or suggestions for content that had not been included in the questionnaire.

### Pilot cross-sectional survey

The pilot cross-sectional survey was performed with a convenience sample of patients with breast cancer in two rounds at a tertiary hospital in Xi’an. The inclusion criteria were being female, aged 18 years or older, and having received a breast cancer diagnosis. Patients with other malignant tumors, severe latent or active infections, cognitive disorders, or psychiatric disorders were excluded.

The questionnaire comprised two sections. The first section assessed socio-demographic variables (i.e., age, education level, marital status, and employment status); the second section comprised phase I of the BISQ-BC (Fig. 1) with the items in a randomized order. Each item was rated on a five-point Likert scale (1 = *strongly disagree*, 5 = *strongly agree*), with higher total scores representing greater effects of body image on the patients. Given that the BISQ-BC is self-reported, patients who could read/write completed the questionnaire by themselves; however, patients who struggled with reading/writing received the interviewer-administered survey, and their responses were recorded by the interviewer verbatim.

### Data analyses

#### Delphi technique data

In each Delphi round, experts were asked to rate each statement according to its importance to the questionnaire using the five-point Likert scale mentioned above. Moreover, they were invited to comment on each item. The items were retained with a coefficient of variation (CV) of ≤0.25 [36] among all experts. Kendall’s W test was used to confirm the relevance of experts’ responses for all items [37]. After each round, the quantitative and qualitative results, and the proposed alterations (i.e., items that should remain or be removed or altered, as well as potential additional items to be added) were discussed among research team members. Feedback after the first round was provided to the Delphi panel via an anonymous summary of the results together with the modified questionnaire and a request for them to evaluate the latter.

#### Pilot cross-sectional survey data

Using these cross-sectional data, we performed further item selection and revision through analyses of the internal consistency (Cronbach’s α) and the convergent and discriminant validity (measured by Spearman’s rho). A Cronbach’s α of ≥0.70 was considered to indicate acceptable reliability [38]. Within a specific subscale, any item that, when deleted, resulted in a higher Cronbach’s α (i.e., a difference of >0.1) was removed from the scale to increase the internal consistency [39].

Regarding convergent validity, a correlation of ≥0.40 between the item and the subscale it is hypothesized to be a part of was indicative of good convergent validity [40]. In contrast, an item had good discriminant validity if it had a stronger correlation with the subscale it was hypothesized to be a part of than with the other subscales [41]. The included items met the criteria that the (1) correlation coefficient between this item and the subscale that it belongs to is greater than 0.40 and (2) it is greater than any of the correlations between this item and the other subscales. The revised items met the former of these two criteria, but not the latter. Removed items failed to meet either criterion.

A database was constructed using EpiData 3.1, and all data were double-entered by two data managers to avoid any possible data-entry errors. Statistical analyses were performed using IBM SPSS Statistics 22.0 (IBM Corp., Armonk, NY). A *p*-value < .05 (two-tailed) was considered significant.

### Ethical considerations

The study received ethical approval from the Biomedical Ethics Committee of Xi’an Jiaotong University Health Science Centre (No. 2015–170). Written informed consent was obtained from each recruited patient before the questionnaire survey.

## Results

### Item pool

The initial item pool comprised 53 items describing the aspects of body image (i.e., body-image-related self-cognition as well as the change in behavior, arm functioning, sexual activity, role, and psychological and social status). All items were further screened through in-depth discussion among the researchers, which led to 13 items being excluded: because of tautology (*n* = 9) or because they were not suitable for the Chinese culture (*n* = 4). The body image aspects were organized into seven subscales in accordance with the characteristics and theoretical framework of body image in breast cancer settings. Phase I of the BISQ-BC ultimately comprised seven subscales with 40 items (Additional file 1; Fig. 1).

### First-round Delphi and pilot cross-sectional survey

Twenty-five eligible experts were recruited in the first round of the Delphi and provided effective responses (Table 1; Fig. 1). Eight items were excluded because their CVs were >0.25. The remaining 32 items all had CVs ≤ 0.25 (Kendall’s W = 0.313, *p* < .001; Additional file 2).

**Table 1:** Characteristics of the Delphi panel experts and breast cancer patients

Participants	Characteristics	*n* (%)
Delphi panel experts (*n* = 25)	Age (yrs) (mean ± SD)	42.20 ± 8.90 (range: 29–61)
	Professional title	
	Professor	14 (56.0)
	Non-professor	11 (44.0)
	Occupation	
	Faculty	8 (32.0)
	Clinical staff	17 (68.0)
	Education level	
	Doctor	9 (36.0)
	Master	9 (36.0)
	Bachelor	7 (28.0)
Patients with Breast cancer (first round) (*n* = 20)	Age (yrs) (mean ± SD)	49.55 ± 10.01 (range: 27–68)
	Education level	
	Primary and below	2 (10.0)
	Secondary	7 (35.0)
	Tertiary	11 (55.0)
	Marital status	
	Single	0 (0.0)
	Married	20 (100.0)
	Employment status	
	Employed	16 (80.0)
	Retired	4 (20.0)
Patients with Breast cancer (second round) (*n* = 50)	Age (yrs) (mean ± SD)	48.44 ± 9.98 (range: 27–73)
	Education level	
	Primary and below	9 (18.0)
	Secondary	12 (24.0)
	Tertiary	29 (58.0)
	Marital status	
	Single	1 (2.0)
	Married	49 (98.0)
	Employment status	
	Employed	46 (92.0)
	Retired	4 (8.0)

A random sample of 20 patients with breast cancer completed the first round of the pilot survey (Table 1; Fig. 1). Only two subscales had acceptable Cronbach’s α values (i.e., body-image-related behavior change: 0.77; body-image-related psychological change: 0.84). The remaining five subscales had lower Cronbach’s α values (i.e., body-image-related self-cognition: 0.51; body-image-related arm change: 0.39; body-image-related sexual activity change: 0.59; body-image-related role change: 0.63; body-image-related social change: 0.47) (Additional file 3).

The validity analysis revealed that the hypothesized item-subscale correlations for all items in all seven subscales were ≥0.40. However, ten items (i.e., *feeling other people are looking at my chest; my arm feels normal; body image change makes me lose my feminine charm; trying to avoid close body contact with others (*e.g.*, embrace); body image change influences my role transformations in family, work, and society; caring about treatment-related body image change; feeling comfortable on body image while exercising; body image change controls my body; disappointment about my current body image;* and *participating in routine activity as usual*) needed to be revised because these correlations were not much higher than the correlations between the item and the other subscales were. One item (i.e., *thinking that certain parts of my body should be hidden*) needed to be removed because its correlation was <0.40 (Additional file 4).

According to the experts, the item “*body image change influences my role transformations in family, work, and society*” was removed and revised into two additional items (i.e., *body image change influences my original family role* and *body image change influences my original work/social role*) for clarification. Additionally, an additional item, “*body image change influences my feelings/attitudes on self-appearance*” was recommended and added to the body-image-related psychological change subscale (Additional file 1; Fig. 1).

In total, eight items (i.e., *thinking of my nude self as sexually charming; thinking that certain parts of my body should be hidden; distressed with the appearance of my arm; body image change influences my role transformations in family, work, and society; angry with my own body; satisfied with my vitality after my body image change; body image change controls my body;* and *satisfied with the appearance of my reconstructed breast/prosthesis*) were excluded and three additional items (i.e., *body image change influences my original family role; body image change influences my original work/social role;* and *body image change influences my feelings/attitudes on self-appearance*) were added in the first round following an in-depth discussion among the research team. Therefore, 35 items across seven subscales were included in the second-round survey (Additional file 1; Fig. 1).

### Second-round Delphi and pilot cross-sectional survey

In the second-round Delphi, all 25 experts who responded to the first round returned suitable responses. All items had CVs ≤ 0.25 (Kendall’s W = 0.238, *p* < .001; Additional file 5).

For the cross-sectional survey, 50 novel patients participated (Table 1; Fig. 1). The subscales had improved internal consistency reliability, with two subscales having Cronbach’s α values ranging from 0.84 (body-image-related psychological change) to 0.88 (body-image-related social change). The other five subscales’ Cronbach’s α values were 0.62 (body-image-related arm change), 0.66 (body-image-related sexual activity change), 0.68 (body-image-related role change), 0.69 (body-image-related self-cognition), and 0.69 (body-image-related behavior change) (Additional file 6).

Most of the hypothesized item-scale correlations with the seven subscales were ≥0.40. However, two items (i.e., t*rying to hide my body while changing clothes alone* and *trying to avoid looking directly at the surgical scar*) were excluded because their correlations were <0.40 (Additional file 7).

According to experts, a new open question, “*Having a sex life or not? (yes* or *no). If no, why?*” was suggested and added to the end of the questionnaire as a supplementary question for gathering information about influences of body image change on patients’ sex life (Additional file 1). This question was not involved in the total score calculation. Therefore, the definitive version of the BISQ-BC after the two rounds of testing had seven subscales with 33 items and one open question (Additional file 1; Fig. 1). Please see the English version of the BISQ-BC in Additional file 8.

## Discussion

A self-rating body image questionnaire was developed for assessing body-image-related aspects among patients with breast cancer. The items contained in the established item pool were adjusted regarding body image characteristics [14, 15], the theoretical framework of body image in a breast cancer context [16], a literature review [4, 13, 33, 34], and in-depth discussion among research team members. Combining the theoretical framework with research on body image in breast cancer settings led us to generate seven subscales reflecting body image from the viewpoint of female patients with breast cancer: psychological change, behavior change, arm functioning, sexual activity change, role change, self-cognition, and social change. This final measure specifically addressed the needs and concerns of Chinese mainland female patients with breast cancer by considering their culture, consulting with Chinese specialists working with breast cancer patients (i.e., Delphi method), and conducting a pilot cross-sectional survey with the target population.

The self-cognition regarding body image subscale was designed to reflect the general self-awareness of the patients on their own self-appearance [1, 14]. It involves mind, satisfaction, belief, expression, being sexually charming, and certain parts of body concentration on body image. However, the items, *thinking of my nude self as sexually charming* and *thinking that certain parts of my body should be hidden* were excluded after the first-round survey because the experts considered the two items as less important; the latter also had poor validity. The item *feeling other people are looking at my chest* was moved to the body-image-related psychological change subscale since it reflected more information about psychological alterations.

It has been widely acknowledged that patients with breast cancer show subsequent behavioral changes following a disruption of body image, including concealing their chest, avoiding changing clothes in public dressing rooms, avoid bathing in public showers, fear that other people are looking directly at their scar, and are concerned with the appearance of their chest [4, 13, 14, 16, 34]. All these aspects were contained in the body-image-related behavior change subscale, except for two excluded items after the second-round survey due to unsupported validity assessment. Furthermore, one of the excluded items, *trying to hide my body while changing clothes alone*, was deemed unnecessary by patients since they felt that it is unnecessary to conceal their body while changing clothes alone. The other excluded item, *trying to avoid looking directly at the surgical scar*, was considered as having somewhat malicious connotations and thus led patients to become more anxious about their illness [12].

Given that lymphedema, which is related to breast cancer, is a common and severe, adverse effect following surgery [42], the body-image-related arm functioning subscale was devised to evaluate body image towards arm appearance, including normal arm feelings, satisfied arm appearance, and the influences of arm swelling and pain on daily living. After the first-round survey, *distressed with the appearance of my arm* was excluded based on the recommendation of experts in that it may be not suitable to those patients who have bilateral breast cancer.

Sexual activity change is widely known as the most common adverse consequence following body image impairments in patients with breast cancer [43]. The related changing activities in sexual life were assessed in the body-image-related sexual activity change subscale (e.g., a loss of feminine charm, avoiding close body contact, covering breasts during sex, sexual confidence/desire, and sex life quality). The items addressing these aspects showed valid results, except for one item: *trying to avoid close body contact with others (*e.g.*, embrace)*, which moved to the body-image-related behavior change subscale since it is more likely a behavior alteration.

Based on published reports, patients with breast cancer also experience role changes after suffering from body image impairments including premature termination from work; inability to do preferred things; and role transformations in the family, at work, and in society [44]. The items belonging to this subscale were appropriate; however, the item *body image change influences my role transformations in family, work, and society* was revised to two additional items (i.e., *body image change influences my original family role* and *body image change influences my original work/social role*) to clarify the point.

Since the body image concept involves perceptions, thoughts, and emotions [1], psychological change has been reported as a key issue following body image alterations [45]. In the subscale of body-image-related psychological change, feelings such as being concerned, comfortable, distressed, angry, satisfied, disappointed, and worried about body image were included. After the first-round survey, four items were excluded from this subscale, with two (i.e., *angry with my own body* and *satisfied with my vitality after my body image change*) being regarded as unimportant by experts, one (i.e., *body image change controls my body*) being considered as difficult to understand by patients, and one (i.e., *satisfied with the appearance of my reconstructed breast/prosthesis*) being suggested as not appropriate for those who do not receive reconstructive surgery.

An additional item, *body image change influences my feelings/attitudes on self-appearance*, was recommended by experts and added to this subscale to evaluate the feelings/attitudes toward self-appearance following body image disturbances. It also had acceptable psychometric results in the second-round survey. Additionally, the item *caring about treatment-related body image change* was moved to the body-image-related behavior change subscale due to a higher correlation between them. Another item, *feeling comfortable with my body image while exercising* was revised to *feeling uncomfortable about my body image* and moved to the body-image-related role change subscale because of the higher correlation between them.

The last subscale, body-image-related social change, was devised to assess the influences of body image impairments on social change of patients with breast cancer [16]. After the first-round survey, the items *trying to avoid participating in social activity* and *limiting social activity due to body image change* had acceptable psychometric properties. However, their descriptions were not easy understood by patients; therefore, they were revised to *trying to avoid participating in social activity due to body image change* and *having to limit social activity due to body image change*, respectively. Both were well validated in the second-round survey. Due to a higher correlation between the item *participating in routine activity as usual* and the body-image-related role-change subscale, this item was rearranged to that subscale and revised as *I cannot participate in routine activity as usual due to body image change* to clarify the point.

Following the experts’ recommendation, a new open question, “*Having a sex life or not? (yes* or *no). If no, why?”* was added to the end of the BISQ-BC. It was designed to obtain more information about impacts of body image alterations on patients’ sex life. This question is just used as a qualitative item and will not be included in the total score calculation.

This study had a major limitation. Given the small sample size of patients with breast cancer in both rounds of the pilot cross-sectional survey, we could only use internal consistency reliability and convergent/discriminant validity for item selection. Other item screening methods such as factor analysis should be conducted in the future with a larger sample size. Furthermore, if Cronbach’s α values of 0.70 and above are deemed acceptable, the internal consistency reliability of five of the subscales (i.e., self-cognition, change in behavior, arm functioning, sexual activity, and role) need to be further tested with larger sample sizes as their Cronbach’s α range was unsatisfactory (i.e., 0.62–0.69).

## Conclusions

Drawing on the characteristics and theoretical framework of body image in breast cancer settings, the BISQ-BC was developed via combining the Delphi technique with an expert panel and a pilot cross-sectional survey. The findings provide evidence of a suitable tool for body image evaluation in Chinese mainland female patients with breast cancer. Studies with larger sample sizes should be conducted to validate this questionnaire in this patient population.

## Additional files

Additional file 1: Summary of the Item Selection Procedure and Corresponding Revisions. (DOC 112 kb)

Additional file 2: Results from the Delphi Technique (Round 1). (DOC 74 kb)

Additional file 3: Results for Cronbach’s α (Round 1). (DOC 63 kb)

Additional file 4: Results of Convergent and Discriminant Validity (Round 1). (DOC 84 kb)

Additional file 5: Results from the Delphi Technique (Round 2). (DOC 62 kb)

Additional file 6: Results for Cronbach’s α (Round 2). (DOC 58 kb)

Additional file 7: Results of Convergent and Discriminant Validity (Round 2). (DOC 78 kb)

Additional file 8: Body Image Self-rating Questionnaire for Breast Cancer (BISQ-BC). (DOC 79 kb)

## Abbreviations

BBIS: Breast and Body Image Scale
BIAQ: Body Image Avoidance Questionnaire
BIBCQ: Body Image After Breast Cancer Questionnaire
BIPIS: Body Image Psychological Inflexibility Scale
BIRS: Body Image and Relationships Scale
BIS: Body Image Scale
BISQ-BC: Body Image Self-rating Questionnaire for Breast Cancer
CV: Coefficient of variation
SABIS: Sexual Adjustment and Body Image Scale
