# Supplementary material for: Development of the body image self-rating questionnaire for breast cancer (BISQ-BC) for Chinese mainland patients
Source: BMC Cancer. 2018 Jan 4;18:19. doi: 10.1186/s12885-017-3865-5 (PMC5753569; doi:10.1186/s12885-017-3865-5)
Supplement: Supplementary file 1 — Summary of the Item Selection Procedure and Corresponding Revisions. (DOC 112 kb) [file 12885_2017_3865_MOESM1_ESM.doc]

Additional file 1 Summary of the item selection procedure and corresponding revisions

| Abbreviated item content of BISQ-BC | Round 1 survey | | | |  | Round 2 survey | | | |  | Final decision^‡^ |
| --- | --- | --- | --- | --- | --- | --- | --- | --- | --- | --- | --- |
|  | Delphi | Reliability | Validity | Screen^†^ |  | Delphi | Reliability | Validity | Screen^†^ |  |  |
| **Body-image-related self-cognition (BI-SCo)** |  |  |  |  |  |  |  |  |  |  |  |
| 1. Caring about my body image | √ | √ | √ | Stay |  | √ | √ | √ | Stay |  | Stay |
| 2. I am satisfied with my body image | √ | √ | √ | Stay |  | √ | √ | √ | Stay |  | Stay |
| 3. Thinking of my body image as attractive |  | √ | √ | Stay |  | √ | √ | √ | Stay |  | Stay |
| 4. Showing my body image via dress and hair style changes | √ | √ | √ | Stay |  | √ | √ | √ | Stay |  | Stay |
| 5. Thinking of my nude self as sexually charming |  | √ | √ | Remove |  |  |  |  |  |  | Remove |
| 6. Thinking that certain parts of my body should be hidden |  | √ |  | Remove |  |  |  |  |  |  | Remove |
| 7. Feeling other people are looking at my chest | √ |  | Revise | Move to BI-PC |  | √ | √ | √ | Stay |  | Stay and move to BI-PC |
| **Body-image-related behaviour change (BI-BC)** |  |  |  |  |  |  |  |  |  |  |  |
| 8. Trying to hide my body especially the breasts | √ | √ | √ | Stay |  | √ | √ | √ | Stay |  | Stay |
| 9. Avoiding changing clothes in the public dressing room | √ | √ | √ | Stay |  | √ | √ | √ | Stay |  | Stay |
| 10. Avoiding taking bath in the public shower room | √ | √ | √ | Stay |  | √ | √ | √ | Stay |  | Stay |
| 11. Trying to hide my body while changing clothes alone |  | √ | √ | Stay |  | √ | √ |  | Remove |  | Remove |
| 12. Trying to avoid others focusing on my body | √ | √ | √ | Stay |  | √ | √ | √ | Stay |  | Stay |
| 13. Checking the appearance of my chest repeatedly | √ | √ | √ | Stay |  | √ | √ | √ | Stay |  | Stay |
| 14. Trying to avoid looking directly at the surgical scar | √ | √ | √ | Stay |  | √ | √ |  | Remove |  | Remove |
| **Body-image-related arm change (BI-AC)** |  |  |  |  |  |  |  |  |  |  |  |
| 15. My arm feels normal | √ | √ | Revise | Stay |  | √ | √ | √ | Stay |  | Stay |
| 16. I am satisfied with the appearance of my arm | √ | √ | √ | Stay |  | √ | √ | √ | Stay |  | Stay |
| 17. Distressed with the appearance of my arm |  | √ | √ | Remove |  |  |  |  |  |  | Remove |
| 18. Arm swelling and pain influence my routine life | √ | √ | √ | Stay |  | √ | √ | √ | Stay |  | Stay |
| **Body-image-related sexual activity change (BI-SAC)** |  |  |  |  |  |  |  |  |  |  |  |
| 19. Body image change makes me lose my feminine charm | √ | √ | Revise | Stay |  | √ | √ | √ | Stay |  | Stay |
| 20. Trying to avoid close body contact with others (e.g., embrace) | √ |  | Revise | Move to BI-BC |  | √ | √ | √ | Stay |  | Stay and move to BI-BC |
| 21. I cover my breasts during sexual activity | √ | √ | √ | Stay |  | √ | √ | √ | Stay |  | Stay |
| 22. Body image change influences my sexual confidence/desire | √ | √ | √ | Stay |  | √ | √ | √ | Stay |  | Stay |
| 23. Body image change influences my sexual life quality | √ | √ | √ | Stay |  | √ | √ | √ | Stay |  | Stay |
| **Body-image-related role change (BI-RC)** |  |  |  |  |  |  |  |  |  |  |  |
| 24. Giving up job due to body image change | √ | √ | √ | Stay |  | √ | √ | √ | Stay |  | Stay |
| 25. I cannot do as I please due to body image changes |  | √ | √ | Stay |  | √ | √ | √ | Stay |  | Stay |
| 26. Body image change influences my role transformations in family, work and society | √ | √ | Remove & revise | Remove & revise to another two new items |  |  |  |  |  |  |  |
| New: Body image change influences my original family role |  |  |  |  |  | √ | √ | √ | Stay |  | Stay |
| New: Body image change influences my original work/social role |  |  |  |  |  | √ | √ | √ | Stay |  | Stay |
| **Body-image-related psychological change (BI-PC)** |  |  |  |  |  |  |  |  |  |  |  |
| 27. Caring about treatment-related body image change | √ | √ | Revise | Move to BI-BC |  | √ | √ | √ | Stay |  | Stay and move to BI-BC |
| 28. Feeling comfortable with my body image while exercising  (Revise: Feeling uncomfortable about my body image) | √ | √ | Revise | Move to BI-RC |  | √ | √ | √ | Stay |  | Stay and move to BI-RC |
| 29. My body feels like it is “breaking down” | √ | √ | √ | Stay |  | √ | √ | √ | Stay |  | Stay |
| 30. Angry with my own body |  | √ | √ | Remove |  |  |  |  |  |  | Remove |
| 31. Satisfied with my vitality after my body image change |  | √ | √ | Remove |  |  |  |  |  |  | Remove |
| 32. Body image change controls my body | √ | √ | Revise | Remove |  |  |  |  |  |  | Remove |
| 33. My breasts are not symmetrical in other people’s eyes | √ | √ | √ | Stay |  | √ | √ | √ | Stay |  | Stay |
| 34. Disappointment about my current body image | √ | √ | Revise | Stay |  | √ | √ | √ | Stay |  | Stay |
| 35. Satisfied with the appearance of my reconstructed breast/prosthesis | √ | √ | √ | Remove |  |  |  |  |  |  | Remove |
| 36. Worrying about relapse while facing the surgical scar | √ | √ | √ | Stay |  | √ | √ | √ | Stay |  | Stay |
| 37. Worrying about health status while facing the surgical scar | √ | √ | √ | Stay |  | √ | √ | √ | Stay |  | Stay |
| New: Body image change influences my feelings/attitudes on self-appearance |  |  |  |  |  | √ | √ | √ | Stay |  | Stay |
| **Body-image-related social change (BI-SC)** |  |  |  |  |  |  |  |  |  |  |  |
| 38. Trying to avoid participating in social activity  (Revise: Trying to avoid participating in social activity due to body image change) | √ | √ | √ | Revision |  | √ | √ | √ | Stay |  | Stay |
| 39. Limiting social activity due to body image change  (Revise: Having to limit social activity due to body image change) | √ | √ | √ | Revision |  | √ | √ | √ | Stay |  | Stay |
| 40. Participating in routine activity as usual  (Revise: I cannot participate in routine activity as usual due to body image change) | √ |  | Revise | (Move to BI-RC) |  | √ | √ | √ | Stay |  | Stay and move to BI-RC |
| A new open question: | | | | | | | | | | | |
| Having a sex life or not? (1) Yes (2) No | | | | | | | | | | | |
| If no, why? | | | | | | | | | | | |

^†^ The item screening was based on experts’ recommendations, statistical analysis results, and in-depth discussions between researchers. ^‡^ The final decision was made following in-depth discussions between researchers. BISQ-BC: Body Image Self-rating Questionnaire for Breast Cancer.
