# Supplementary material for: Development of the body image self-rating questionnaire for breast cancer (BISQ-BC) for Chinese mainland patients
Source: BMC Cancer. 2018 Jan 4;18:19. doi: 10.1186/s12885-017-3865-5 (PMC5753569; doi:10.1186/s12885-017-3865-5)
Supplement: Supplementary file 2 — Results from the Delphi Technique (Round 1). (DOC 74 kb) [file 12885_2017_3865_MOESM2_ESM.doc]

Additional file 2 Results Delphi round 1 (N = 25)

| Abbreviated item content of BISQ-BC | Mean | SD | CV | Result^†^ |
| --- | --- | --- | --- | --- |
| **Body-image-related self-cognition (BI-SCo)** |  |  |  |  |
| 1. Caring about my body image | 4.16 | 0.69 | 0.17 | Stay |
| 2. I am satisfied with my body image | 3.92 | 0.91 | 0.23 | Stay |
| 3. Thinking of my body image as attractive | 3.56 | 1.12 | 0.31 | Remove |
| 4. Showing my body image via dress and hair style changes | 3.88 | 0.83 | 0.21 | Stay |
| 5. Thinking of my nude self as sexually charming | 2.92 | 1.19 | 0.41 | Remove |
| 6. Thinking that certain parts of my body should be hidden | 3.84 | 0.99 | 0.26 | Remove |
| 7. Feeling other people are looking at my chest | 3.76 | 0.93 | 0.25 | Stay |
| **Body-image-related behaviour change (BI-BC)** |  |  |  |  |
| 8. Trying to hide my body especially the breasts | 4.76 | 0.52 | 0.11 | Stay |
| 9. Avoiding changing clothes in the public dressing room | 4.80 | 0.50 | 0.10 | Stay |
| 10. Avoiding taking bath in the public shower room | 4.76 | 0.44 | 0.09 | Stay |
| 11. Trying to hide my body while changing clothes alone | 3.52 | 1.00 | 0.28 | Remove |
| 12. Trying to avoid others focusing on my body | 4.32 | 0.63 | 0.15 | Stay |
| 13. Checking the appearance of my chest repeatedly | 3.80 | 0.87 | 0.23 | Stay |
| 14. Trying to avoid looking directly at the surgical scar | 4.12 | 0.78 | 0.19 | Stay |
| **Body-image-related arm change (BI-AC)** |  |  |  |  |
| 15. My arm feels normal | 4.36 | 0.70 | 0.16 | Stay |
| 16. I am satisfied with the appearance of my arm | 3.96 | 0.89 | 0.22 | Stay |
| 17. Distressed with the appearance of my arm | 3.76 | 0.97 | 0.26 | Remove |
| 18. Arm swelling and pain influence my routine life | 4.08 | 0.86 | 0.21 | Stay |
| **Body-image-related sexual activity change (BI-SAC)** |  |  |  |  |
| 19. Body image change makes me lose my feminine charm | 4.56 | 0.65 | 0.14 | Stay |
| 20. Trying to avoid close body contact with others (e.g., embrace) | 4.32 | 0.63 | 0.15 | Stay |
| 21. I cover my breasts during sexual activity | 4.40 | 0.71 | 0.16 | Stay |
| 22. Body image change influences my sexual confidence/desire | 4.48 | 0.59 | 0.13 | Stay |
| 23. Body image change influences my sexual life quality | 4.52 | 0.65 | 0.14 | Stay |
| **Body-image-related role change (BI-RC)** |  |  |  |  |
| 24. Giving up job due to body image change | 3.72 | 0.89 | 0.24 | Stay |
| 25. I cannot do as I please due to body image changes | 3.56 | 1.00 | 0.28 | Remove |
| 26. Body image change influences my role transformations in family, work, and society | 3.88 | 0.88 | 0.23 | Stay |
| **Body-image-related psychological change (BI-PC)** |  |  |  |  |
| 27. Caring about treatment-related body image change | 4.60 | 0.58 | 0.13 | Stay |
| 28. Feeling comfortable with my body image while exercising | 3.84 | 0.90 | 0.23 | Stay |
| 29. My body feels like it is “breaking down” | 4.00 | 0.76 | 0.19 | Stay |
| 30. Angry with my own body | 3.56 | 1.12 | 0.31 | Remove |
| 31. Satisfied with my vitality after my body image change | 3.36 | 0.99 | 0.29 | Remove |
| 32. Body image change controls my body | 3.43 | 0.81 | 0.24 | Stay |
| 33. My breasts are not symmetrical in other people’s eyes | 4.76 | 0.52 | 0.11 | Stay |
| 34. Disappointment about my current body image | 4.36 | 0.70 | 0.16 | Stay |
| 35. Satisfied with the appearance of my reconstructed breast/prosthesis | 4.20 | 0.58 | 0.14 | Stay |
| 36. Worrying about relapse while facing the surgical scar | 4.48 | 0.59 | 0.13 | Stay |
| 37. Worrying about health status while facing the surgical scar | 4.52 | 0.51 | 0.11 | Stay |
| **Body-image-related social change (BI-SC)** |  |  |  |  |
| 38. Trying to avoid participating in social activity | 4.24 | 0.60 | 0.14 | Stay |
| 39. Limiting social activity due to body image change | 4.12 | 0.73 | 0.18 | Stay |
| 40. Participating in routine activity as usual | 4.12 | 0.88 | 0.21 | Stay |

Kendall’s W = 0.313, χ^2^ = 305.32, *P* < 0.001.

BISQ-BC: Body Image Self-rating Questionnaire for Breast Cancer.

SD: standard deviation.

CV: coefficient of variation.

^†^ All stay items meet the criteria of CV ≤ 0.25.
