# Supplementary material for: Development of the body image self-rating questionnaire for breast cancer (BISQ-BC) for Chinese mainland patients
Source: BMC Cancer. 2018 Jan 4;18:19. doi: 10.1186/s12885-017-3865-5 (PMC5753569; doi:10.1186/s12885-017-3865-5)
Supplement: Supplementary file 3 — Results for Cronbach’s α (Round 1). (DOC 63 kb) [file 12885_2017_3865_MOESM3_ESM.doc]

Additional file 3 Results Cronbach’s α round 1 (N = 20)

| Abbreviated item content of BISQ-BC | Cronbach’s α | Result^†^ |
| --- | --- | --- |
| **Body-image-related self-cognition (BI-SCo)** | **0.51** |  |
| 1. Caring about my body image | 0.35 | Stay |
| 2. I am satisfied with my body image | 0.33 | Stay |
| 3. Thinking of my body image as attractive | 0.26 | Stay |
| 4. Showing my body image via dress and hair style changes | 0.40 | Stay |
| 5. Thinking of my nude self as sexually charming | 0.54 | Stay |
| 6. Thinking that certain parts of my body should be hidden | 0.58 | Stay |
| 7. Feeling other people are looking at my chest | 0.65 | Remove |
| **Body-image-related behaviour change (BI-BC)** | **0.77** |  |
| 8. Trying to hide my body especially the breasts | 0.69 | Stay |
| 9. Avoiding changing clothes in the public dressing room | 0.75 | Stay |
| 10. Avoiding taking bath in the public shower room | 0.76 | Stay |
| 11. Trying to hide my body while changing clothes alone | 0.76 | Stay |
| 12. Trying to avoid others focusing on my body | 0.70 | Stay |
| 13. Checking the appearance of my chest repeatedly | 0.74 | Stay |
| 14. Trying to avoid looking directly at the surgical scar | 0.76 | Stay |
| **Body-image-related arm change (BI-AC)** | **0.39** |  |
| 15. My arm feels normal | 0.43 | Stay |
| 16. I am satisfied with the appearance of my arm | 0.34 | Stay |
| 17. Distressed with the appearance of my arm | 0.29 | Stay |
| 18. Arm swelling and pain influence my routine life | 0.22 | Stay |
| **Body-image-related sexual activity change (BI-SAC)** | **0.59** |  |
| 19. Body image change makes me lose my feminine charm | 0.63 | Stay |
| 20. Trying to avoid close body contact with others (e.g., embrace) | 0.68 | Stay |
| 21. I cover my breasts during sexual activity | 0.50 | Stay |
| 22. Body image change influences my sexual confidence/desire | 0.39 | Stay |
| 23. Body image change influences my sexual life quality | 0.41 | Stay |
| **Body-image-related role change (BI-RC)** | **0.63** |  |
| 24. Giving up job due to body image change | 0.49 | Stay |
| 25. I cannot do as I please due to body image changes | 0.45 | Stay |
| 26. Body image change influences my role transformations in family, work, and society | 0.66 | Stay |
| **Body-image-related psychological change (BI-PC)** | **0.84** |  |
| 27. Caring about treatment-related body image change | 0.84 | Stay |
| 28. Feeling comfortable with my body image while exercising | 0.87 | Stay |
| 29. My body feels like it is “breaking down” | 0.81 | Stay |
| 30. Angry with my own body | 0.81 | Stay |
| 31. Satisfied with my vitality after my body image change | 0.84 | Stay |
| 32. Body image change controls my body | 0.83 | Stay |
| 33. My breasts are not symmetrical in other people’s eyes | 0.80 | Stay |
| 34. Disappointment about my current body image | 0.82 | Stay |
| 35. Satisfied with the appearance of my reconstructed breast/prosthesis | 0.83 | Stay |
| 36. Worrying about relapse while facing the surgical scar | 0.82 | Stay |
| 37. Worrying about health status while facing the surgical scar | 0.82 | Stay |
| **Body-image-related social change (BI-SC)** | **0.47** |  |
| 38. Trying to avoid participating in social activity | -0.03 | Stay |
| 39. Limiting social activity due to body image change | 0.23 | Stay |
| 40. Participating in routine activity as usual | 0.72 | Remove |

^†^ Within a specific subscale, any item that, when deleted, resulted in a higher Cronbach’s α (i.e., the difference > 0.1) was removed from the scale.

BISQ-BC: Body Image Self-rating Questionnaire for Breast Cancer.
