# Supplementary material for: Development of the body image self-rating questionnaire for breast cancer (BISQ-BC) for Chinese mainland patients
Source: BMC Cancer. 2018 Jan 4;18:19. doi: 10.1186/s12885-017-3865-5 (PMC5753569; doi:10.1186/s12885-017-3865-5)
Supplement: Supplementary file 4 — Results of Convergent and Discriminant Validity (Round 1). (DOC 84 kb) [file 12885_2017_3865_MOESM4_ESM.doc]

Additional file 4 Results Convergent validity^a^ and discriminant validity^b^ round 1 (Spearman’s rho) (N = 20)

| Abbreviated item content of BISQ-BC | BI-SCo | BI-BC | BI-AC | BI-SAC | BI-RC | BI-PC | BI-SC | Result^†^ |
| --- | --- | --- | --- | --- | --- | --- | --- | --- |
| **Body-image-related self-cognition (BI-SCo)** |  |  |  |  |  |  |  |  |
| 1. Caring about my body image | **0.68** | -0.17 | -0.01 | -0.46 | -0.42 | -0.38 | -0.27 | Stay |
| 2. I am satisfied with my body image | **0.74** | -0.50 | -0.21 | 0.56 | -0.61 | -0.71 | -0.74 | Stay |
| 3. Thinking of my body image as attractive | **0.84** | -0.23 | -0.31 | -0.20 | -0.54 | -0.53 | -0.56 | Stay |
| 4. Showing my body image via dress and hair style changes | **0.69** | -0.18 | -0.37 | 0.04 | -0.58 | -0.26 | -0.54 | Stay |
| 5. Thinking of my nude self as sexually charming | **0.41** | -0.01 | 0.08 | -0.53 | -0.22 | -0.21 | -0.10 | Stay |
| 6. Thinking that certain parts of my body should be hidden | **0.19** | 0.17 | 0.19 | 0.12 | -0.06 | -0.02 | -0.02 | Remove |
| 7. Feeling other people are looking at my chest | **0.04** | 0.09 | 0.13 | 0.51 | 0.39 | 0.41 | 0.33 | Revise |
| **Body-image-related behaviour change (BI-BC)** |  |  |  |  |  |  |  |  |
| 8. Trying to hide my body especially the breasts | -0.08 | **0.84** | 0.14 | 0.17 | 0.40 | 0.15 | 0.24 | Stay |
| 9. Avoiding changing clothes in the public dressing room | -0.70 | **0.51** | -0.10 | 0.45 | 0.36 | 0.26 | 0.36 | Stay |
| 10. Avoiding taking bath in the public shower room | -0.39 | **0.51** | -0.06 | 0.50 | 0.10 | 0.13 | 0.26 | Stay |
| 11. Trying to hide my body while changing clothes alone | 0.02 | **0.57** | -0.23 | 0.56 | 0.19 | 0.08 | -0.11 | Stay |
| 12. Trying to avoid others focusing on my body | -0.31 | **0.80** | 0.20 | 0.17 | 0.42 | 0.34 | 0.37 | Stay |
| 13. Checking the appearance of my chest repeatedly | -0.15 | **0.60** | -0.01 | 0.36 | 0.28 | 0.35 | 0.31 | Stay |
| 14. Trying to avoid looking directly at the surgical scar | 0.31 | **0.54** | 0.26 | 0.11 | -0.06 | -0.06 | -0.002 | Stay |
| **Body-image-related arm change (BI-AC)** |  |  |  |  |  |  |  |  |
| 15. My arm feels normal | -0.23 | 0.24 | **0.60** | 0.08 | 0.62 | 0.44 | 0.57 | Revise |
| 16. I am satisfied with the appearance of my arm | 0.17 | 0.002 | **0.50** | 0.28 | -0.05 | 0.20 | 0.21 | Stay |
| 17. Distressed with the appearance of my arm | -0.03 | 0.05 | **0.52** | -0.08 | -0.28 | 0.02 | 0.16 | Stay |
| 18. Arm swelling and pain influence my routine life | -0.41 | 0.17 | **0.66** | 0.007 | 0.28 | 0.18 | 0.44 | Stay |
| **Body-image-related sexual activity change (BI-SAC)** |  |  |  |  |  |  |  |  |
| 19. Body image change makes me lose my feminine charm | -0.42 | 0.24 | 0.27 | **0.64** | 0.44 | 0.68 | 0.60 | Revise |
| 20. Trying to avoid close body contact with others (e.g., embrace) | -0.10 | 0.41 | 0.23 | **0.44** | 0.24 | 0.48 | 0.33 | Revise |
| 21. I cover my breasts during sexual activity | -0.20 | 0.42 | -0.03 | **0.58** | 0.19 | 0.02 | 0.14 | Stay |
| 22. Body image change influences my sexual confidence/desire | 0.05 | 0.23 | -0.16 | **0.69** | -0.04 | 0.08 | -0.04 | Stay |
| 23. Body image change influences my sexual life quality | 0.07 | 0.23 | -0.16 | **0.68** | -0.06 | 0.08 | -0.04 | Stay |
| **Body-image-related role change (BI-RC)** |  |  |  |  |  |  |  |  |
| 24. Giving up job due to body image change | -0.24 | 0.32 | -0.04 | 0.10 | **0.56** | 0.18 | 0.32 | Stay |
| 25. I cannot do as I please due to body image changes | -0.19 | 0.19 | 0.07 | -0.10 | **0.74** | 0.05 | 0.22 | Stay |
| 26. Body image change influences my role transformations in family, work, and society | -0.50 | 0.34 | 0.42 | 0.42 | **0.76** | 0.81 | 0.72 | Revise |
| **Body-image-related psychological change (BI-PC)** |  |  |  |  |  |  |  |  |
| 27. Caring about treatment-related body image change | -0.27 | 0.59 | 0.20 | 0.21 | 0.44 | **0.43** | 0.34 | Revise |
| 28. Feeling comfortable with my body image while exercising | -0.38 | 0.14 | 0.22 | 0.02 | 0.47 | **0.14** | 0.26 | Revise |
| 29. My body feels like it is “breaking down” | -0.26 | 0.28 | 0.26 | 0.39 | 0.42 | **0.69** | 0.63 | Stay |
| 30. Angry with my own body | -0.27 | 0.15 | 0.20 | 0.56 | 0.30 | **0.74** | 0.64 | Stay |
| 31. Satisfied with my vitality after my body image change | 0.06 | -0.01 | 0.10 | 0.24 | 0.08 | **0.48** | 0.09 | Stay |
| 32. Body image change controls my body | -0.24 | 0.47 | 0.37 | 0.39 | 0.54 | **0.54** | 0.62 | Revise |
| 33. My breasts are not symmetrical in other people’s eyes | -0.58 | 0.26 | 0.14 | 0.57 | 0.49 | **0.90** | 0.65 | Stay |
| 34. Disappointment about my current body image | -0.41 | 0.28 | 0.07 | 0.77 | 0.34 | **0.76** | 0.58 | Revise |
| 35. Satisfied with the appearance of my reconstructed breast/prosthesis | -0.42 | -0.01 | 0.30 | 0.04 | 0.51 | **0.62** | 0.47 | Stay |
| 36. Worrying about relapse while facing the surgical scar | -0.39 | -0.17 | 0.20 | 0.05 | 0.49 | **0.71** | 0.64 | Stay |
| 37. Worrying about health status while facing the surgical scar | -0.39 | -0.21 | 0.23 | 0.20 | 0.44 | **0.71** | 0.69 | Stay |
| **Body-image-related social change (BI-SC)** |  |  |  |  |  |  |  |  |
| 38. Trying to avoid participating in social activity | -0.22 | 0.54 | 0.46 | 0.40 | 0.48 | 0.48 | **0.75** | Stay |
| 39. Limiting social activity due to body image change | -0.51 | 0.09 | 0.51 | 0.46 | 0.35 | 0.64 | **0.74** | Stay |
| 40. Participating in routine activity as usual | -0.28 | 0.11 | 0.32 | -0.04 | 0.56 | 0.42 | **0.55** | Revise |

^a^ Convergent validity: the hypothesized item-scale correlations ≥ 0.40 are in bold.

^b^ Discriminant validity: the hypothesized item-scale correlation was higher than the alternative ones.

^†^ Items designated as “stay” met the criteria that the (1) correlation coefficient between this item and the subscale to which it belongs is greater than 0.40 and (2) it is greater than any of the correlations between this item and the other subscales; “change” items met the former of these two criteria, but not the latter; and “remove” items failed to meet either criterion.

BISQ-BC: Body Image Self-rating Questionnaire for Breast Cancer.
