# Supplementary material for: Development of the body image self-rating questionnaire for breast cancer (BISQ-BC) for Chinese mainland patients
Source: BMC Cancer. 2018 Jan 4;18:19. doi: 10.1186/s12885-017-3865-5 (PMC5753569; doi:10.1186/s12885-017-3865-5)
Supplement: Supplementary file 5 — Results from the Delphi Technique (Round 2). (DOC 62 kb) [file 12885_2017_3865_MOESM5_ESM.doc]

Additional file 5 Results Delphi round 2 (N = 25)

| Abbreviated item content of BISQ-BC | Mean | SD | CV | Result |
| --- | --- | --- | --- | --- |
| **Body-image-related self-cognition (BI-SCo)** |  |  |  |  |
| 1. Caring about my body image | 4.48 | 0.59 | 0.13 | Stay |
| 2. I am satisfied with my body image | 4.32 | 0.56 | 0.13 | Stay |
| 3. Thinking of my body image as attractive | 3.92 | 0.70 | 0.18 | Stay |
| 4. Showing my body image via dress and hair style changes | 4.44 | 0.71 | 0.16 | Stay |
| **Body-image-related behaviour change (BI-BC)** |  |  |  |  |
| 5. Caring about treatment-related body image change | 4.76 | 0.52 | 0.11 | Stay |
| 6. Trying to avoid close body contact with others (e.g., embrace) | 4.64 | 0.64 | 0.14 | Stay |
| 7. Trying to hide my body especially the breasts | 4.84 | 0.37 | 0.08 | Stay |
| 8. Avoiding changing clothes in the public dressing room | 4.80 | 0.41 | 0.09 | Stay |
| 9. Avoiding taking bath in the public shower room | 4.80 | 0.41 | 0.09 | Stay |
| 10. Trying to hide my body while changing clothes alone | 3.92 | 0.86 | 0.22 | Stay |
| 11. Trying to avoid others focusing on my body | 4.80 | 0.41 | 0.09 | Stay |
| 12. Checking the appearance of my chest repeatedly | 4.20 | 0.76 | 0.18 | Stay |
| 13. Trying to avoid looking directly at the surgical scar | 4.48 | 0.65 | 0.15 | Stay |
| **Body-image-related arm change (BI-AC)** |  |  |  |  |
| 14. My arm feels normal | 4.72 | 0.61 | 0.13 | Stay |
| 15. I am satisfied with the appearance of my arm | 4.44 | 0.65 | 0.15 | Stay |
| 16. Arm swelling and pain influence my routine life | 4.36 | 0.70 | 0.16 | Stay |
| **Body-image-related sexual activity change (BI-SAC)** |  |  |  |  |
| 17. Body image change makes me lose my feminine charm | 4.72 | 0.54 | 0.11 | Stay |
| 18. I cover my breasts during sexual activity | 4.72 | 0.61 | 0.13 | Stay |
| 19. Body image change influences my sexual confidence/desire | 4.72 | 0.46 | 0.10 | Stay |
| 20. Body image change influences my sexual life quality | 4.80 | 0.41 | 0.09 | Stay |
| **Body-image-related role change (BI-RC)** |  |  |  |  |
| 21. Giving up job due to body image change | 4.00 | 0.82 | 0.21 | Stay |
| 22. I cannot do as I please due to body image changes | 4.04 | 0.84 | 0.21 | Stay |
| 23. Feeling uncomfortable about my body image | 4.56 | 0.71 | 0.16 | Stay |
| 24. Cannot participate in routine activity as usual | 4.32 | 0.69 | 0.16 | Stay |
| 25. Body image change influences my original family role | 4.24 | 0.72 | 0.17 | Stay |
| 26. Body image change influences my original work/social role | 4.40 | 0.76 | 0.17 | Stay |
| **Body-image-related psychological change (BI-PC)** |  |  |  |  |
| 27. Feeling other people are looking at my chest | 4.56 | 0.65 | 0.14 | Stay |
| 28. My body feels like it is “breaking down” | 4.52 | 0.65 | 0.14 | Stay |
| 29. Body image change influences my feelings/attitudes on self-appearance | 4.88 | 0.33 | 0.07 | Stay |
| 30. My breasts are not symmetrical in other people’s eyes | 4.80 | 0.50 | 0.10 | Stay |
| 31. Disappointment about my current body image | 4.48 | 0.65 | 0.15 | Stay |
| 32. Worrying about relapse while facing the surgical scar | 4.80 | 0.50 | 0.10 | Stay |
| 33. Worrying about health status while facing the surgical scar | 4.76 | 0.44 | 0.09 | Stay |
| **Body-image-related social change (BI-SC)** |  |  |  |  |
| 34. Trying to avoid participating in social activity due to body image change | 4.60 | 0.50 | 0.11 | Stay |
| 35. Having to limit social activity due to body image change | 4.48 | 0.59 | 0.13 | Stay |

Kendall’s W = 0.238, χ^2^ = 202.06, *P* < 0.001.

BISQ-BC: Body Image Self-rating Questionnaire for Breast Cancer.

SD: standard deviation.

CV: coefficient of variation.

^†^ All stay items meet the criteria of CV < 0.25.
