# Supplementary material for: Development of the body image self-rating questionnaire for breast cancer (BISQ-BC) for Chinese mainland patients
Source: BMC Cancer. 2018 Jan 4;18:19. doi: 10.1186/s12885-017-3865-5 (PMC5753569; doi:10.1186/s12885-017-3865-5)
Supplement: Supplementary file 6 — Results for Cronbach’s α (Round 2). (DOC 58 kb) [file 12885_2017_3865_MOESM6_ESM.doc]

Additional file 6 Results Cronbach’s α round 2 (N = 50)

| Abbreviated item content of BISQ-BC | Cronbach’s α | Result^†^ |
| --- | --- | --- |
| **Body-image-related self-cognition (BI-SCo)** | **0.69** |  |
| 1. Caring about my body image | 0.68 | Stay |
| 2. I am satisfied with my body image | 0.65 | Stay |
| 3. Thinking of my body image as attractive | 0.46 | Stay |
| 4. Showing my body image via dress and hair style changes | 0.66 | Stay |
| **Body-image-related behaviour change (BI-BC)** | **0.69** |  |
| 5. Caring about treatment-related body image change | 0.66 | Stay |
| 6. Trying to avoid close body contact with others (e.g., embrace) | 0.70 | Stay |
| 7. Trying to hide my body especially the breasts | 0.63 | Stay |
| 8. Avoiding changing clothes in the public dressing room | 0.63 | Stay |
| 9. Avoiding taking bath in the public shower room | 0.66 | Stay |
| 10. Trying to hide my body while changing clothes alone | 0.71 | Stay |
| 11. Trying to avoid others focusing on my body | 0.62 | Stay |
| 12. Checking the appearance of my chest repeatedly | 0.67 | Stay |
| 13. Trying to avoid looking directly at the surgical scar | 0.72 | Stay |
| **Body-image-related arm change (BI-AC)** | **0.62** |  |
| 14. My arm feels normal | 0.42 | Stay |
| 15. I am satisfied with the appearance of my arm | 0.46 | Stay |
| 16. Arm swelling and pain influence my routine life | 0.67 | Stay |
| **Body-image-related sexual activity change (BI-SAC)** | **0.66** |  |
| 17. Body image change makes me lose my feminine charm | 0.66 | Stay |
| 18. I cover my breasts during sexual activity | 0.70 | Stay |
| 19. Body image change influences my sexual confidence/desire | 0.51 | Stay |
| 20. Body image change influences my sexual life quality | 0.48 | Stay |
| **Body-image-related role change (BI-RC)** | **0.68** |  |
| 21. Giving up job due to body image change | 0.71 | Stay |
| 22. I cannot do as I please due to body image changes | 0.64 | Stay |
| 23. Feeling uncomfortable about my body image | 0.63 | Stay |
| 24. Cannot participate in routine activity as usual | 0.59 | Stay |
| 25. Body image change influences my original family role | 0.64 | Stay |
| 26. Body image change influences my original work/social role | 0.62 | Stay |
| **Body-image-related psychological change (BI-PC)** | **0.84** |  |
| 27. Feeling other people are looking at my chest | 0.82 | Stay |
| 28. My body feels like it is “breaking down” | 0.82 | Stay |
| 29. Body image change influences my feelings/attitudes on self-appearance | 0.81 | Stay |
| 30. My breasts are not symmetrical in other people’s eyes | 0.84 | Stay |
| 31. Disappointment about my current body image | 0.82 | Stay |
| 32. Worrying about relapse while facing the surgical scar | 0.83 | Stay |
| 33. Worrying about health status while facing the surgical scar | 0.82 | Stay |
| **Body-image-related social change (BI-SC)** | **0.88** |  |
| 34. Trying to avoid participating in social activity due to body image change | - | Stay |
| 35. Having to limit social activity due to body image change | - | Stay |

^†^ Within a specific subscale, any item that, when deleted, resulted in a higher Cronbach’s α (i.e., the difference > 0.1) was removed from the scale.

BISQ-BC: Body Image Self-rating Questionnaire for Breast Cancer.
