# Supplementary material for: Development of the body image self-rating questionnaire for breast cancer (BISQ-BC) for Chinese mainland patients
Source: BMC Cancer. 2018 Jan 4;18:19. doi: 10.1186/s12885-017-3865-5 (PMC5753569; doi:10.1186/s12885-017-3865-5)
Supplement: Supplementary file 7 — Results of Convergent and Discriminant Validity (Round 2). (DOC 78 kb) [file 12885_2017_3865_MOESM7_ESM.doc]

Additional file 7 Results Convergent validity^a^ and discriminant validity^b^ round 2 (Spearman’s rho) (N = 50)

| Abbreviated item content of BISQ-BC | BI-SCo | BI-BC | BI-AC | BI-SAC | BI-RC | BI-PC | BI-SC | Result^†^ |
| --- | --- | --- | --- | --- | --- | --- | --- | --- |
| **Body-image-related self-cognition (BI-SCo)** |  |  |  |  |  |  |  |  |
| 1. Caring about my body image | **0.54** | 0.23 | 0.10 | 0.32 | 0.08 | -0.09 | -0.01 | Stay |
| 2. I am satisfied with my body image | **0.73** | 0.34 | -0.03 | 0.15 | -0.07 | -0.07 | 0.06 | Stay |
| 3. Thinking of my body image as attractive | **0.86** | 0.24 | -0.07 | 0.06 | 0.23 | -0.16 | 0.08 | Stay |
| 4. Showing my body image via dress and hair style changes | **0.60** | 0.08 | 0.07 | 0.06 | 0.005 | 0.01 | 0.05 | Stay |
| **Body-image-related behaviour change (BI-BC)** |  |  |  |  |  |  |  |  |
| 5. Caring about treatment-related body image change | 0.24 | **0.58** | 0.12 | 0.35 | 0.07 | 0.18 | 0.32 | Stay |
| 6. Trying to avoid close body contact with others (e.g., embrace) | -0.06 | **0.46** | 0.11 | 0.12 | 0.07 | -0.04 | 0.12 | Stay |
| 7. Trying to hide my body especially the breasts | 0.43 | **0.70** | 0.18 | 0.54 | 0.12 | 0.26 | 0.28 | Stay |
| 8. Avoiding changing clothes in the public dressing room | 0.29 | **0.65** | 0.04 | 0.49 | 0.10 | 0.20 | 0.24 | Stay |
| 9. Avoiding taking bath in the public shower room | -0.08 | **0.47** | -0.01 | 0.29 | 0.06 | 0.07 | 0.15 | Stay |
| 10. Trying to hide my body while changing clothes alone | -0.06 | **0.32** | -0.04 | 0.08 | 0.006 | -0.27 | -0.13 | Remove |
| 11. Trying to avoid others focusing on my body | 0.23 | **0.66** | -0.02 | 0.36 | 0.14 | 0.05 | 0.07 | Stay |
| 12. Checking the appearance of my chest repeatedly | 0.32 | **0.57** | 0.16 | 0.49 | 0.30 | 0.34 | 0.41 | Stay |
| 13. Trying to avoid looking directly at the surgical scar | 0.18 | **0.35** | 0.09 | 0.14 | -0.007 | 0.02 | 0.08 | Remove |
| **Body-image-related arm change (BI-AC)** |  |  |  |  |  |  |  |  |
| 14. My arm feels normal | 0.14 | 0.27 | **0.78** | 0.40 | 0.31 | 0.31 | 0.32 | Stay |
| 15. I am satisfied with the appearance of my arm | 0.06 | 0.09 | **0.71** | 0.18 | 0.12 | 0.13 | 0.06 | Stay |
| 16. Arm swelling and pain influence my routine life | -0.14 | 0.13 | **0.74** | 0.21 | 0.06 | 0.08 | -0.14 | Stay |
| **Body-image-related sexual activity change (BI-SAC)** |  |  |  |  |  |  |  |  |
| 17. Body image change makes me lose my feminine charm | 0.05 | 0.37 | 0.31 | **0.70** | 0.56 | 0.57 | 0.35 | Stay |
| 18. I cover my breasts during sexual activity | 0.28 | 0.30 | 0.24 | **0.54** | 0.07 | -0.05 | -0.15 | Stay |
| 19. Body image change influences my sexual confidence/desire | 0.21 | 0.60 | 0.12 | **0.75** | 0.33 | 0.40 | 0.24 | Stay |
| 20. Body image change influences my sexual life quality | 0.10 | 0.20 | 0.22 | **0.71** | 0.41 | 0.47 | 0.20 | Stay |
| **Body-image-related role change (BI-RC)** |  |  |  |  |  |  |  |  |
| 21. Giving up job due to body image change | -0.19 | -0.16 | -0.12 | 0.12 | **0.41** | 0.27 | 0.07 | Stay |
| 22. I cannot do as I please due to body image changes | 0.03 | 0.18 | 0.05 | 0.30 | **0.64** | 0.33 | 0.30 | Stay |
| 23. Feeling uncomfortable about my body image | -0.09 | 0.15 | 0.26 | 0.38 | **0.68** | 0.53 | 0.27 | Stay |
| 24. Cannot participate in routine activity as usual | -0.02 | 0.21 | 0.29 | 0.36 | **0.71** | 0.57 | 0.45 | Stay |
| 25. Body image change influences my original family role | 0.10 | -0.14 | 0.27 | 0.16 | **0.57** | 0.20 | 0.11 | Stay |
| 26. Body image change influences my original work/social role | 0.15 | 0.21 | 0.11 | 0.16 | **0.64** | 0.25 | 0.30 | Stay |
| **Body-image-related psychological change (BI-PC)** |  |  |  |  |  |  |  |  |
| 27. Feeling other people are looking at my chest | -0.15 | 0.26 | 0.36 | 0.51 | 0.41 | **0.74** | 0.47 | Stay |
| 28. My body feels like it is “breaking down” | -0.09 | 0.12 | 0.05 | 0.30 | 0.55 | **0.71** | 0.53 | Stay |
| 29. Body image change influences my feelings/attitudes on self-appearance | -0.05 | 0.14 | 0.16 | 0.49 | 0.51 | **0.78** | 0.44 | Stay |
| 30. My breasts are not symmetrical in other people’s eyes | 0.001 | -0.12 | 0.32 | 0.27 | 0.33 | **0.61** | 0.18 | Stay |
| 31. Disappointment about my current body image | 0.04 | 0.33 | 0.11 | 0.61 | 0.66 | **0.75** | 0.45 | Stay |
| 32. Worrying about relapse while facing the surgical scar | -0.24 | -0.04 | 0.13 | 0.17 | 0.15 | **0.65** | 0.51 | Stay |
| 33. Worrying about health status while facing the surgical scar | -0.18 | 0.16 | 0.09 | 0.22 | 0.28 | **0.72** | 0.67 | Stay |
| **Body-image-related social change (BI-SC)** |  |  |  |  |  |  |  |  |
| 34. Trying to avoid participating in social activity due to body image change | 0.10 | 0.37 | 0.08 | 0.25 | 0.39 | 0.63 | **0.95** | Stay |
| 35. Having to limit social activity due to body image change | 0.11 | 0.23 | 0.12 | 0.24 | 0.42 | 0.59 | **0.94** | Stay |

^a^ Convergent validity: the hypothesized item-scale correlations ≥ 0.40 are in bold.

^b^ Discriminant validity: the hypothesized item-scale correlation was higher than the alternative ones.

^†^ Items designated as “stay” met the criteria that the (1) correlation coefficient between this item and the subscale to which it belongs is greater than 0.40 and (2) it is greater than any of the correlations between this item and the other subscales; “change” items met the former of these two criteria, but not the latter; and “remove” items failed to meet either criterion.

BISQ-BC: Body Image Self-rating Questionnaire for Breast Cancer.
