# Supplementary material for: Development of the body image self-rating questionnaire for breast cancer (BISQ-BC) for Chinese mainland patients
Source: BMC Cancer. 2018 Jan 4;18:19. doi: 10.1186/s12885-017-3865-5 (PMC5753569; doi:10.1186/s12885-017-3865-5)
Supplement: Supplementary file 8 — Body Image Self-rating Questionnaire for Breast Cancer (BISQ-BC). (DOC 79 kb) [file 12885_2017_3865_MOESM8_ESM.doc]

Additional file 8 Body Image Self-rating Questionnaire for Breast Cancer (BISQ-BC)

| Abbreviated item content of BISQ-BC | Strongly  disagree | Disagree | Neither disagree  nor agree | Agree | Strongly  agree |
| --- | --- | --- | --- | --- | --- |
| **Body-image-related self-cognition (BI-SCo)** |  |  |  |  |  |
| 1. Caring about my body image | 1 | 2 | 3 | 4 | 5 |
| 2. I am satisfied with my body image | 1 | 2 | 3 | 4 | 5 |
| 3. Thinking of my body image as attractive | 1 | 2 | 3 | 4 | 5 |
| 4. Showing my body image via dress and hair style changes | 1 | 2 | 3 | 4 | 5 |
| **Body-image-related behaviour change (BI-BC)** |  |  |  |  |  |
| 5. Caring about treatment-related body image change | 1 | 2 | 3 | 4 | 5 |
| 6. Trying to avoid close body contact with others (e.g., embrace) | 1 | 2 | 3 | 4 | 5 |
| 7. Trying to hide my body especially the breasts | 1 | 2 | 3 | 4 | 5 |
| 8. Avoiding changing clothes in the public dressing room | 1 | 2 | 3 | 4 | 5 |
| 9. Avoiding taking bath in the public shower room | 1 | 2 | 3 | 4 | 5 |
| 10. Trying to avoid others focusing on my body | 1 | 2 | 3 | 4 | 5 |
| 11. Checking the appearance of my chest repeatedly | 1 | 2 | 3 | 4 | 5 |
| **Body-image-related arm change (BI-AC)** |  |  |  |  |  |
| 12. My arm feels normal | 1 | 2 | 3 | 4 | 5 |
| 13. I am satisfied with the appearance of my arm | 1 | 2 | 3 | 4 | 5 |
| 14. Arm swelling and pain influence my routine life | 1 | 2 | 3 | 4 | 5 |
| **Body-image-related sexual activity change (BI-SAC)** |  |  |  |  |  |
| 15. Body image change makes me lose my feminine charm | 1 | 2 | 3 | 4 | 5 |
| 16. I cover my breasts during sexual activity | 1 | 2 | 3 | 4 | 5 |
| 17. Body image change influences my sexual confidence/desire | 1 | 2 | 3 | 4 | 5 |
| 18. Body image change influences my sexual life quality | 1 | 2 | 3 | 4 | 5 |
| **Body-image-related role change (BI-RC)** |  |  |  |  |  |
| 19. Giving up job due to body image change | 1 | 2 | 3 | 4 | 5 |
| 20. I cannot do as I please due to body image changes | 1 | 2 | 3 | 4 | 5 |
| 21. Feeling uncomfortable about my body image | 1 | 2 | 3 | 4 | 5 |
| 22. I cannot participate in routine activity as usual due to body image change | 1 | 2 | 3 | 4 | 5 |
| 23. Body image change influences my original family role | 1 | 2 | 3 | 4 | 5 |
| 24. Body image change influences my original work/social role | 1 | 2 | 3 | 4 | 5 |
| **Body-image-related psychological change (BI-PC)** |  |  |  |  |  |
| 25. Feeling other people are looking at my chest | 1 | 2 | 3 | 4 | 5 |
| 26. My body feels like it is “breaking down” | 1 | 2 | 3 | 4 | 5 |
| 27. Body image change influences my feelings/attitudes on self-appearance | 1 | 2 | 3 | 4 | 5 |
| 28. My breasts are not symmetrical in other people’s eyes | 1 | 2 | 3 | 4 | 5 |
| 29. Disappointment about my current body image |  |  |  |  |  |
| 30. Worrying about relapse while facing the surgical scar | 1 | 2 | 3 | 4 | 5 |
| 31. Worrying about health status while facing the surgical scar | 1 | 2 | 3 | 4 | 5 |
| **Body-image-related social change (BI-SC)** |  |  |  |  |  |
| 32. Trying to avoid participating in social activity due to body image change | 1 | 2 | 3 | 4 | 5 |
| 33. Having to limit social activity due to body image change | 1 | 2 | 3 | 4 | 5 |
| A new open question: | | | | | |
| Having a sex life or not? (1) Yes (2) No | | | | | |
| If no, why? | | | | | |
